# Dose Escalation Can Enhance the Therapeutic Potential of Radial Extracorporeal Shock-Wave Therapy in the Treatment of Plantar Fasciitis in Runners

**DOI:** 10.3390/medicina60050766

**Published:** 2024-05-06

**Authors:** Sebastian Szajkowski, Jarosław Pasek, Grzegorz Cieślar

**Affiliations:** 1 Faculty of Medical and Social Sciences, Warsaw Medical Academy of Applied Sciences, 8 Rydygiera St., 01-793 Warszawa, Poland; sebastianszajkowski@wp.pl; 2 Collegium Medicum im dr Władysława Biegańskiego, Jan Długosz University in Częstochowa, 13/15 Armii Krajowej St., 42-200 Częstochowa, Poland; 3 Department of Internal Medicine, Angiology and Physical Medicine, Faculty of Medical Sciences in Zabrze, Medical University of Silesia in Katowice, 15 Stefana Batorego St., 41-902 Bytom, Poland; cieslar1@o2.pl

**Keywords:** shock-wave therapy, calcaneal spur, myofascial stiffness, musculoskeletal system, treatment

## Abstract

*Background and Objectives:* Treatment of chronic plantar fasciitis is challenging given that there are various of available treatment options with no clear gold standard. The aim of the study was to examine the dose-escalation effect of rESWT on the biomechanical parameters of the plantar fascia and pain ailments. *Materials and Methods:* In the experimental group (*n* = 30), the intensity of the shock wave was increased every two subsequent treatment sessions. In the control group (*n* = 32), the treatment parameters were not changed. In both groups, six treatments were performed, with two treatment sessions a week. In order to assess the biomechanical parameters of the plantar fascia, myotonometric measurements were performed. The pain intensity was assessed using the Visual Analog Scale (VAS). *Results:* The tension of the plantar fascia attachment in the experimental group decreased from 27.69 ± 2.06 [Hz] before treatment to 26.29 ± 1.69 [Hz] after treatment (*p* = 0.009) and to 26.03 ± 2.15 [Hz] 1 month after the beginning of treatment (*p* = 0.003). In the control group, the frequency results did not change significantly (*p* > 0.05). Flexibility increased in both groups. The test results before treatment and 1 month after the beginning of the treatment showed statistical significance in the experimental group (*p* = 0.001) vs. (*p* = 0.002) in the control group. The differences were not statistically significant between groups (*p* > 0.05). The assessment of pain intensity carried out 1 month after the end of treatment in the experimental group amounted to 3.14 ± 2.28 points, which was statistically significantly lower compared to that in the control group, where it amounted to 5.14 ± 1.92 points. (*p* < 0.001). *Conclusions:* The use of rESWT performed with an increasing intensity of impact during subsequent treatment procedures demonstrated greater effectiveness in improving the biomechanical parameters of the plantar fascia and was also more effective in reducing the pain ailments. Our results are encouraging. The dose escalation in the treatment cycle is worth considering. To prove that this method of treatment is more effective, a randomized controlled trial should be carried out on a representative sample.

## 1. Introduction

Plantar fasciitis is the most common non-traumatic cause of heel pain. It accounts for 11–15% of musculoskeletal disorders in the foot area that require professional care [1]. It is the subject of a significant number of studies due to its complex pathophysiology and varying effectiveness of treatment attempts. Most of these studies focus on older, physically inactive people. The proposed risk factors in their case, such as increased BMI, the coexistence of systemic diseases, the development of the disease and its course, and susceptibility to the type of treatment used, are different than in the case of younger people who regularly practice various sports disciplines as amateurs, including running. Among runners, the most important risk factors are those that disrupt the proper biomechanics of the foot and increase the tension of the plantar fascia. These include lowering of the longitudinal arch of the foot, pronation in the lower ankle joint, and deficit of dorsiflexion in the upper ankle joint. Modifiable peri-training factors include a too-rapid increase of load in training units, inappropriate footwear, and increased body weight [2,3].

The pain occurring in the insertion of the plantar fascia in the area of the heel bone in cases of runners develops as a result of the accumulation of micro-injuries related to repeated overload during training, i.e., excessive eccentric loading of the insertion of the aponeurosis, with subsequent degeneration and chronic inflammation [4]. Plantar fasciitis is diagnosed in 42% of middle-distance runners and 25% of long-distance runners [5]. Patients report stabbing and burning heel pain as a result of weight bearing [6]. The symptoms are particularly severe in the morning after waking up and after a long period of immobility. The pain usually alleviates after undertaking physical activity [7]. On physical examination, tenderness on palpation has been revealed in the heel bone. The diagnosis should be confirmed by an imaging test. X-ray or ultrasound examinations are the tests most often performed. The prognosis for recovery remains unclear, and the treatment itself is often ineffective. However, it has been shown that the disease is self-limiting, and pain symptoms subside regardless of the treatment used, even over a period of up to 1 year. On the other hand, the typically chronic course of the disease predisposes to a secondary occurrence of symptoms in 50% of people within 5 years of making the diagnosis. The duration and severity of pain also significantly compromise the quality of life [6,8].

Due to the assumed pathophysiology of the disease, various alternative therapeutic interventions are applied to its treatment. The most common ones include manual therapy, exercises, heel-lifting orthoses, kinesio taping, shock wave and ultrasound, corticosteroid injections, NSAIDs, and PRP (autologous blood-derived injections such as platelet-rich plasma). However, each of the above-mentioned methods has limitations concerning its effectiveness. So far, there has been no standard procedure established that ensures effective treatment [9,10,11,12,13,14]. There are promising results from randomized controlled trials, providing various treatment regimens for patients with plantar fasciitis. However, according to the literature, no consensus has been reached yet as to what treatment should be recommended in these cases. An additional difficulty comes from the fact that, in most studies, their authors have decided to apply combined therapies (e.g., shock-wave therapy linked with other rehabilitation treatments provided in parallel). The methodology of many studies remains unclear and makes it considerably difficult to compare the treatment effects achieved. There are no high-quality reports available in the literature regarding the therapeutic use of shock waves in the treatment of plantar fasciopathy. One of the reasons for the low quality of the research performed in this area is the absence of an objective, quantitative method for assessing the results of treatment [15,16,17].

The study reported here assessed the rESWT radial shock-wave therapy, which is a commonly applied treatment from the field of physical medicine used for treating plantar fasciitis and symptomatic heel spurs. rESWT therapy is an effective method of stimulating tissue healing. rESWT uses pneumatic waves generated by an air compressor and released through a cap at the end of the applicator. A bullet hits the applicator and the pressure wave which is generated is transferred to the tissues. The procedure requires the application of ultrasound gel as a coupling agent [18,19,20,21,22,23].

In the course of such non-invasive treatment, high-amplitude sound waves are generated, which focus on the tissue being treated [18,19]. As shown by the meta-analysis made by Babatunde et al., it is a treatment that is highly effective compared to other treatment methods [21]. These observations have been confirmed by the study of Fouda et al. [22]. The benefits of shock-wave treatment were also confirmed in patients over a long-term observation period of 3 months after the end of the treatment cycle [23].

An objective, repeatable method of measurement is necessary to evaluate the effectiveness of plantar fasciitis treatment. These requirements are met by myotonometry. This method provides quantitative data that allow for making an objective assessment of the biomechanical properties of aponeurosis during treatment. Studies conducted previously have shown that the MyotonPRO (Myoton AS, Tallinn, Estonia) device proves to be reliable in the assessment of mechanical properties of plantar fascia [24,25], as well as other tendons [26,27,28].

Such properties include tension, stiffness, and elasticity [25]. The need to find the most effective treatment parameters on the one hand and to provide methods that guarantee precise monitoring of treatment results on the other hand remains fully justified in order to develop the best care protocol for a patient with plantar fasciitis [12,29].

According to the authors of the study reported here, more frequent studies should be undertaken involving the population of physically active people and individuals practicing sports in order to get a better understanding of the etiology and diagnostic criteria of plantar fasciitis. An understanding of how plantar fasciitis manifests itself in the population of people practicing sports may translate into subsequent and appropriate use of therapeutic interventions and appropriate physiotherapy for such people, ultimately leading to the improvement of treatment results [30].

The aim of the study was to investigate the effect of increasing doses of the rESWT shock-wave therapy in subsequent treatments of the biomechanical parameters of the plantar fascia and for pain ailments of runners with plantar fasciitis.

## 2. Material and Methods

### 2.1. Participants

The study included 62 amateurs who had been practicing medium- (<5 km) and long-distance (>5 km) running at least twice a week for at least 5 years and were diagnosed with plantar fasciitis. The rESWT radial shock-wave therapy was used as treatment. Participants were randomly assigned to two groups using the random number generator in Microsoft Excel. In group 1, the experimental group (*n* = 30; 18 women and 12 men, 10 persons practicing medium-distance and 20 persons practicing long-distance running), the treatment parameters were changed during treatment. The intensity of shock-wave therapy was increased every 2 subsequent treatment sessions based on increasing pain tolerance. In group 2, the control group (*n* = 32; 19 women and 13 men, 10 persons practicing medium-distance running and 22 persons practicing long-distance running), the shock-wave treatment parameters were not changed during treatment.

The inclusion criteria for participation in the study were as follows: age between 45 and 60 years, heel spur visible on X-ray or ultrasound image, pain in the heel bone when body weight was applied while standing, pain experienced when taking the first steps in the morning and after prolonged immobility during the day, tenderness on palpation in the place where the plantar fascia are attached to the heel bone, duration of symptoms of at least a month, and recurrence of pain. The exclusion criteria were as follows: previous treatment for a heel spur in the period of 2 months preceding the study, pharmacological treatment, including application of corticosteroid injections, physiotherapy, and the coexistence of neurological and rheumatoid diseases.

The patients were requested to refrain from training for at least 1 week before the start of treatment, during it, and for at least one month after the end of the therapeutic cycle until the final assessment of the results of the therapy.

The general characteristics of the study participants, including age, height and weight, length of training history, and duration of symptoms, were collected during the medical interview at the first visit.

The sample size was established by considering the feasibility of conducting comprehensive follow-ups in the case of each patient. G*power software (version 3.1.9.7; Heinrich-Heine-Universität Düsseldorf, Düsseldorf, Germany; (http://www.gpower.hhu.de), accessed on 5 January 2024 [31] was used to determine the power using 2-sided testing, α = 0.05 and β = 0.2. The effect size was 0.94. The power (1-β err prob) was calculated as 0.95. The post hoc calculation was made for the independent *t*-test.

The study was approved by the Bioethics Committee at the Medical University of Mazovia in Warsaw, Poland (approval reference number: 2022/09/MUM-01, approval date: 30 September 2022). The study was conducted in accordance with the guidelines of good clinical practice and the recommendations of the Declaration of Helsinki (1964).

### 2.2. Procedures

#### 2.2.1. Radial Extracorporeal Shock-Wave Therapy—rESWT

During shock-wave treatment procedures, the patients were asked to lie on their stomachs with their knees straight. The foot remained in a natural resting position, outside the couch. The most painful place on the heel bone, corresponding to the place of insertion of the plantar fascia, was located by palpation [32]. In order to properly transmit the energy generated by the device, a coupling gel was used to ensure that the transmitter fits the tissue. The applicator’s transmitter remained in contact with the skin throughout the entire course of the procedure. A typical labile shock-wave application method was used. In group 1 (experimental one), the following treatment parameters were used: continuous pulse-emission mode; frequency of 14 Hz in the 1st and 2nd treatment, then reduced by 2 Hz to 12 Hz in the 3rd and 4th treatment, and to 10 Hz in the 5th and 6th treatment; compressor pressure 2.5 bar (250 kPa) in treatment 1, then increased in each subsequent treatment by 0.5 bar (50 kPa), up to the level of 5 bar (500 kPa) in treatment 6; and the number of blows in treatment 1 amounted to 3000, then it was increased by 500 blows with each subsequent treatment, which means that 5000 blows were administered in the 5th and 6th treatment. In group 2 (control), the following treatment parameters were used: continuous pulse emission mode, frequency of 12 Hz, compressor pressure of 3 bar (300 kPa), and the number of shocks during the treatment was 3000. The same shock-wave parameters were used in each subsequent treatment in the therapeutic cycle.

In both groups, 6 treatments were performed, with the frequency of 2 treatments a week, with a 2–3 day break between treatments. The Impactis M+ radial shock-wave generator (Astar) was used for the purpose of conducting the therapy. In both groups, the TR10-TI titanium transmitter was used for performing the procedures. The energy density at the maximum working pressure was 0.38 mJ/mm^2^, ranging from 0.28 to 0.6 mJ/mm^2^. Therefore, these were treatments with medium energy radial shock-wave therapy (MERSWT). As in the literature, the energy-flux density (EFD) is classified as follows: high energy (>0.6 mJ/mm^2^), medium energy (0.28–0.6 mJ/mm^2^), and low energy (<0.28 mJ/mm^2^) [28,29,30,31]. No other methods of therapy were used during the treatment.

#### 2.2.2. MyotonPRO Examination

Before commencing the myotonometric measurements, the patients had 10 min to rest and relax their feet. The probe of the Myoton PRO device (Myoton AS, Tallinn, Estonia) was placed in a perpendicular position to the skin surface, in the projection of the attachment of the plantar fascia to the heel bone [32,33]. The device was calibrated during the contact of the probe with the examined tissue by applying the initial pressure of 0.18 N. In the next stage, the MyotonPRO generated 5 short mechanical impulses, with a force of 0.4 N and a duration of 15 ms. The accelerometer recorded the oscillations of the examined tissue. Three parameters corresponding to biomechanical stiffness were calculated: (F)—oscillation/vibration frequency [Hz], which determines the tissue tension (tone); (S)—dynamic stiffness (stiffness) [N/m], which characterizes the resistance of tissue to deformation; and (D)—decrement [log], which characterizes the reduction of tissue oscillations and, conversely, describes its elasticity. The measurement results were interpreted as follows: the higher the values of (F)—oscillation/vibration frequency [Hz] and (S)—dynamic stiffness [N/m], the greater the tension and stiffness of the examined tissue. The lower the value of (D)—damping (decrement) [log], the lower the dissipation of mechanical energy during oscillations and the greater the elasticity of the tissue [25,34]. The assessment of treatment was carried out before the start of treatment, after the end of the treatment cycle, and 1 month after the end of treatment.

#### 2.2.3. Assessment of Pain Severity

A visual analog scale, VAS, was used to assess the severity of pain. It is a scale (from 0 to 10) with a 10 cm long graphic section on the other side of the ruler, on which the patient marks the intensity of the pain experienced. After inverting the ruler, it was possible to take the readings, where the value of 0 stands for no pain, and the value of 10 signifies the greatest pain imaginable [35].

All the therapeutic procedures with the use of the rESWT, the measurements taken by means of the MyotonPRO device, and the assessment of pain intensity using the VAS scale were performed by an experienced physiotherapist, trained in the field of myotonometry and performing scientific research.

### 2.3. Statistical Analysis

The Statistica 13 package (Statsoft, Kraków, Poland) was used to analyze the research results. The results were presented using mean values and standard deviation. The nature of the distribution of the studied variables was tested using the Shapiro–Wilk test. A one-way repeated-measures ANOVA and a post hoc Scheffé test were used to examine the statistical significance of the differences between individual days of the experiment in each group separately. Student’s *t*-test was used to test the statistical significance of the differences in the studied parameters between groups. The level of statistical significance was assumed to be *p* < 0.05. For the independent-samples *t*-test, Cohen’s d was used to compute the effect size. An effect size for the repeated-measures ANOVA was calculated via the partial eta squared (η2p).

## 3. Results

The descriptive statistics for the demographic data of age, height, weight, body mass index (BMI), training history, and duration of symptoms for both groups of study participants are presented in Table 1. The groups were homogeneous. No statistically significant differences have been noted.

**Figure 1:**
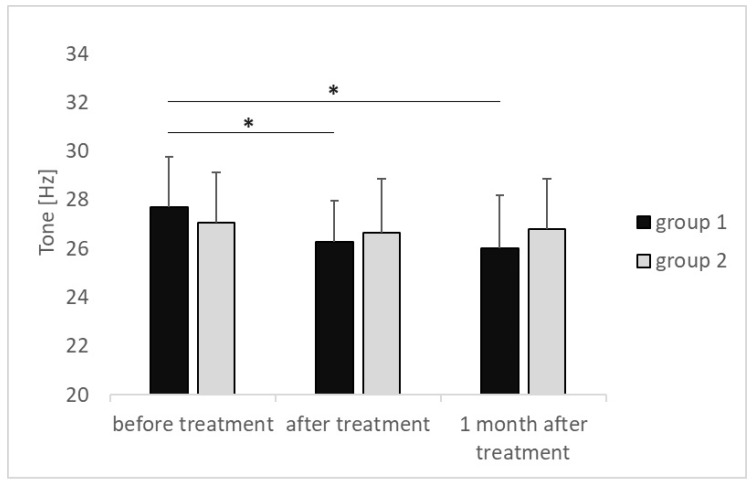
Changes in tone of the calcaneal attachment of the plantar fascia—results of the MyotonPRO measurements at particular time intervals in both groups that were compared. *p* value of <0.05 is represented as *.

The results of the measurements obtained before treatment, immediately after treatment, and 1 month after the end of treatment were compared in group 1 and group 2, respectively. The tension of the plantar fascia attachment in group 1 decreased from 27.69 ± 2.06 [Hz] before treatment to 26.29 ± 1.69 [Hz] after treatment (*p* = 0.009) and decreased further, reaching 26.03 ± 2.15 [Hz] 1 month after the end of treatment (*p* = 0.003); effect size (η2p = 0.001). In group 2, the tension decreased from 27.05 ± 2.06 [Hz] before treatment to 26.67 ± 2.18 [Hz] after treatment and finally increased, reaching 26.79 ± 2.07 [Hz] 1 month after the end of treatment. The tension of the plantar fascia attachment did not undergo any statistically significant changes during the treatment and 1 month after its completion (*p* > 0.05) (Figure 1).

**Figure 2:**
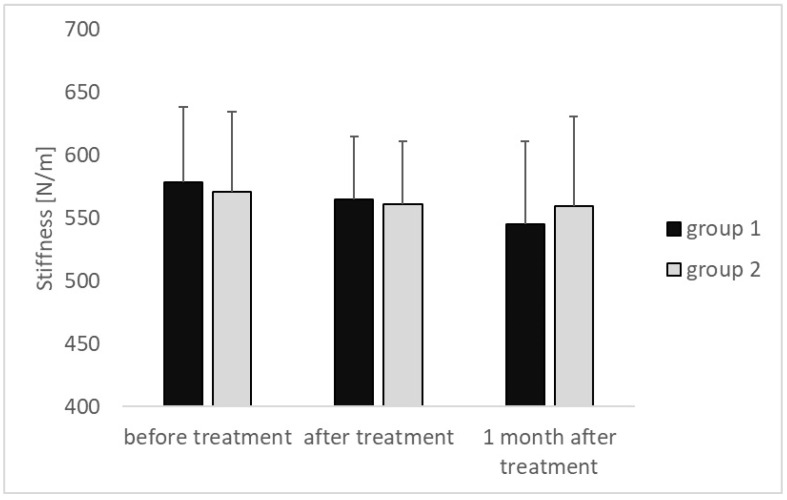
Changes in stiffness of the calcaneal attachment of the plantar fascia—results of the MyotonPRO measurements at particular time intervals for both groups that were compared.

The stiffness of the plantar fascia attachment decreased in both compared groups in each subsequent examination, amounting in group 1 to 578.57 ± 59.72 [N/m] vs. 564.82 ± 49.87 [N/m] vs. 544.82 ± 66.26 [N/m] and in group 2 to 570.86 ± 63.76 [N/m] vs. 561.00 ± 50.14 [N/m] vs. 559.86 ± 71.09 [N/m], respectively. Neither in group 1 nor in group 2 did the observed differences reach the level of statistical significance (*p* > 0.05) (Figure 2).

**Figure 3:**
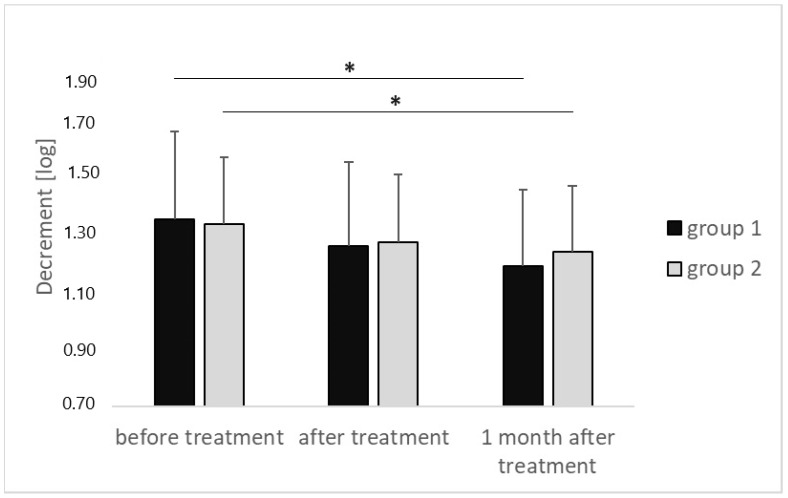
Changes in decrement of the calcaneal attachment of the plantar fascia—results of the MyotonPRO measurements at particular time intervals for both groups that were compared. *p* value of <0.05 is represented as *.

The decrement values, inversely describing flexibility in group 1, decreased at subsequent time intervals, with the difference in values obtained for the measurement performed before the start of treatment amounting to 1.34 ± 0.30 [log] and the values from the measurement performed 1 month after the end of treatment amounting to 1.18 ± 0.26 [log], showing statistical significance (*p* < 0.001); effect size (η2p = 0.002). Similarly, in group 2, the decrement values decreased at subsequent time intervals, with the difference in the value of the measurement performed before the start of treatment amounting to 1.32 ± 0.23 [log] and the value of the measurement performed 1 month after the end of treatment, of 1.23 ± 0.22 [log], showing statistical significance (*p* = 0.002); effect size (η2p = 0.001). (Figure 3).

The values of all myotonometric parameters were also compared between groups 1 and 2 in subsequent examinations, with no statistically significant differences found (*p* > 0.05). In a study conducted 1 month after the end of treatment, tension and stiffness in group 1 were significantly lower and flexibility was significantly higher when compared to group 2.

The intensity of pain measured on the VAS scale before treatment was estimated to amount to the average of 63.8 ± 1.5 [mm] in group 1 and 60.3 ± 1.8 [mm] in group 2, and the difference observed was not statistically significant (*p* = 0.405). The intensity of pain after the series of treatments was slightly lower in group 1, with 45.2 ± 1.68 vs. 49.7 ± 1.57 [mm] in group 2, and the difference observed did not show statistical significance (*p* = 0.271). However, the intensity of pain assessed 1 month after the end of treatment in group 1, which was 31.4 ± 2.28 [mm], was statistically significantly lower compared to group 2, where it amounted to 51.4 ± 1.92 [mm], (*p* < 0.001); effect size (Cohen’s d = 0.94). The pain intensity assessed before treatment, immediately after treatment, and 1 month after the end of treatment was also compared in each group separately. In group 1, the intensity of pain assessed in subsequent measurements was systematically reduced, and the differences in values between subsequent measurements showed statistical significance. In group 2, the intensity of pain after the end of the treatment was statistically significantly lower than before the treatment, while in the study conducted 1 month after the end of the therapeutic cycle, the intensity of pain increased, with no statistical significance compared to the value observed immediately after the end of treatment. The pain-intensity results are presented in Table 2.

## 4. Discussion

The study assessed the impact of increasing the rESWT doses in subsequent treatment procedures of the therapeutic cycle on the effectiveness of treatment of plantar fasciitis in runners. The results were compared with standard procedures performed using constant rESWT values throughout the treatment period. The most important conclusions were as follows. The tension of the plantar fascia attachment in the group in which the dose of rESWT was gradually increased in subsequent treatments decreased in each subsequent myotometric examination. Such results were not obtained for the group in which procedures were performed using constant values for the rESWT parameters. Stiffness decreased and flexibility increased throughout the entire observation period in both groups. However, in the experimental group, the values of tension and stiffness were lower, and the elasticity values were higher at the end of the observation period, i.e., 1 month after the end of treatment. This indicates the greater effectiveness of the experimental method of performing treatments in restoring the correct biomechanical properties of plantar fascia. Better biomechanical parameters of the plantar fascia in the experimental group were accompanied by a lower pain intensity assessed 1 month after the end of treatment. The above observations show that escalating the rESWT doses during the cycle of therapy consisting of six treatments at a frequency of two treatments a week with a 2–3 day break between treatments results in a more effective treatment when compared to standard treatments with constant rESWT doses throughout the entire observation period.

In the available literature, there are no results available of studies using myotonometry for the assessment of the biomechanical parameters of the degenerative and inflamed plantar fascia attachments after treatment with rESWT. To our knowledge, the study reported here is the first one that evaluates the effectiveness of treatment of plantar fasciitis with rESWT by means of myotonometry. In the literature available from the following databases, PubMed, Medline, Cochrane Library, Springer Link, Clinical Trials.gov, and OVID, only two studies had been available before November 2023 that describe the biomechanical properties of plantar fascia using myotonometry, and they both concern healthy individuals. The search terms used in the query were plantar fasciitis, plantar fascia, PF, calcaneal spur and radial extracorporeal shock-wave therapy, rESWT, shock-wave therapy, SWT and myotonometry, or MyotonPro.

The first of the studies found this way is a prospective study assessing the biomechanical properties of plantar fascia in a population of 176 asymptomatic individuals [25]. The plantar fascia was examined medially, not at its attachment to the calcaneal tuberosity. The average voltage-measurement results amounted to 25.94 ± 2.11 [Hz], compared to our results, i.e., 27.69 ± 2.06 [Hz] before treatment and 26.03 ± 2.15 [Hz] after 1 month after the end of treatment. The average stiffness value amounted to 533.18 ± 64.56 [N/m], compared to the values obtained by us in the measurements made before treatment, amounting to 578.57 ± 59.72 [N/m], and after treatment, reaching 544.82 ± 66.26 [N/m]. However, the average value of the decrement, which inversely describes elasticity, was 1.16 ± 0.14 [log], compared to the value of 1.34 ± 0.30 [log] obtained in our study before treatment and that of 1.18 ± 0.26 [log] in the measurement performed 1 month after the end of treatment. The above study concerned the part of the plantar fascia that is expected to be characterized by lower tension and stiffness and to be more flexible compared to the aponeurosis attachment on the heel bone, which is characterized by a higher density of collagen fibers [36].

The observations from our study confirm the occurrence of this regularity in all three parameters measured with the myotonometric test. During the course of treatments using rESWT, as well as in the long-term evaluation of outcomes, i.e., 1 month after the end of treatment, the values of the parameters describing the biomechanical properties of the plantar fascia were similar to those observed in healthy individuals.

The second study assessed 30 healthy participants as regards the impact of knee-joint position (extension and 90 degrees of passive flexion) and ankle-joint position (50 degrees of passive plantar flexion; 25 degrees of passive dorsiflexion; and, similar to the conditions applied in our study: in a neutral position, 0 degrees) on stiffness of the plantar fascia. The stiffness in the area where the aponeurosis is attached to the heel tuberosity was the lowest in plantar flexion and increased successively in the neutral and dorsal flexion positions. The stiffness values measured in all the above ankle-joint settings were, correspondingly, higher with the knee straight than with the knee bent. The proximal attachment of the gastrocnemius muscle appears to be responsible for this, which, as a bi-articular muscle, relaxes when the knee is passively flexed in a relief position [37]. This, in turn, affects the tension and stiffness of the Achilles tendon, which, as shown by Wang JS, significantly affects the biomechanics of the plantar fascia [38]. On the other hand, the soleus muscle has an impact on the resultant tension and stiffness of the Achilles tendon, which also affects the mechanics of the plantar fascia. This muscle, in turn, is a mono-articular muscle, and a 90-degree knee flexion increases its shear modulus in shear-wave elastography [39]. The use of elastography instead of myotonometry in this case resulted from the anatomical location of the soleus muscle, located/positioned under the gastrocnemius muscle. Therefore, the position in which the authors of this study performed the myotonometric examination was an intermediate position of the ankle joint a neutral one, not affecting the stiffness of the aponeurosis. Additionally, a straight position of the knee joint with the subject lying on the stomach—without a roller or wedge under the lower leg—also provided the most reliable conditions for the assessment of the biomechanical parameters of the plantar fascia, eliminating the possibility of additional positional relaxation in this case.

There are more studies that can be found in the available literature that assess the effectiveness of treatment of degenerative and inflammatory changes of the plantar fascia using rESWT, especially in comparison to ultrasound therapy (UD). In a meta-analysis carried out by Li et al. in 2019 five papers were analyzed [16].

Justifying the exclusive use of radial ESWT over alternative modalities, such as focused shock-wave therapy or ultrasound-guided injections, would provide clarity on treatment selection [40].

Although the differences between the study groups were not significant and both treatment methods proved to be effective in the treatment of plantar fasciitis, the VAS score proved to be better in the ESWT group, suggesting that it may be a better alternative in the treatment of plantar fasciitis. It should be noted that the number of high-quality studies included in this meta-analysis was relatively small. A large number of studies carried out on this topic are characterized by a high diversity of the population, a variety of methods for assessing treatment results, and the combining of different treatment methods into one treatment protocol. All this contributes to a high level of heterogeneity among the studies, which significantly complicates the meta-analysis and produces diverse clinical results.

Another meta-analysis carried out (network meta-analysis) [41] compared the effectiveness of alleviating the intensity of pain in the course of plantar fasciitis (assessed using the VAS scale) by using eight different types of therapy, namely nonsteroid anti-inflammatory drugs, corticosteroid injections (CS), autologous whole blood, platelet-rich plasma (PRP), extracorporeal shockwave therapy (ESWT), ultrasound therapy (US), botulinum toxin type A (BTXA), and dry needling (DN). Forty-one prospective or randomized controlled trials were included, comprising a total of 2889 cases. In the 1-month observation period, only one (ESWT) turned out to be more effective than placebo (mean difference = −3.3; 95% credible intervals: [−5.3, −1.1]). There were no statistically significant differences in comparisons regarding 2-month follow-up. At 3-month follow-up, ESWT and corticosteroid injections (CS) were more effective than placebo. In the longest follow-up period of 6 months, only ESWT was rated better than placebo. The authors of the meta-analysis recommend ESWT as the optimal method in the treatment of plantar fasciitis.

The use of rESWT in the treatment of plantar fascia is also recommended by the authors of yet another meta-analysis [11]. It included 19 studies involving a total of 1676 patients with plantar fasciitis. The meta-analysis showed that only rESWT caused a significant reduction in pain compared to placebo in the period from 0 to 6 weeks after treatment (mean difference = 3.67, 95% confidence intervals: (0.31, 6.9). No significant differences were found for periods from 2 to 4 months and from 6 to 12 months after treatment due to wide confidence intervals of 95%. The authors of that study clearly recommend the treatment of plantar fasciitis using rESWT.

In another study, the therapeutic effectiveness of ESWT with the energy density of 0.35 mJ/mm^2^ at 10.5 kV and the total dose of 350 mJ/mm^2^ was examined, i.e., within a range similar to that used by us in the case of patients with treatment-resistant plantar fasciitis lasting >6 months. A 30% reduction in pain intensity on the VAS scale was achieved in 81% of the treated patients after 6 weeks of observation. Ultimately, a satisfactory reduction in pain intensity was achieved for 96% of patients, and it persisted up to 73 months after the end of treatment (data from a telephone survey) [42].

The results of the meta-analysis carried out proved that the use of medium-energy ESWT, regardless of the type of shock-wave generators used, was more effective in terms of pain relief compared to the control group in the period up to the 12th month of observation. However, the effectiveness of low- and high-energy ESWT was uncertain when compared to the control group in terms of the pain intensity assessed on the VAS scale during the observation period, reaching up to 12 months. This is in line with our treatment results obtained with the application of rESWT in the range of the average values of the energy used [17].

To sum up, rESWT is highly effective in the treatment of plantar fasciitis and can be considered the golden standard among conservative treatment methods [43], but the selection of optimal physical parameters remains an open issue and requires continued research with a clear indication of its high quality. The authors of the study reported here also have basic research in mind, because the biological mechanisms of the impact of rESWT in the treatment of musculoskeletal diseases remain, unfortunately, not fully explained. rESWT has an analgesic effect exerted through the mechanism of peripheral nerve stimulation (PNS), which explains the “Gate Control Theory” proposed by Melzack and Wall in 1965, suggesting that applying non-painful stimuli to the low-threshold, non-nociceptive, large-diameter A-beta nerve fibers causes activation of the inhibitors of interneurons, the inhibition of conduction of nociceptive A-delta and C nerve fibers, and discharge in the dorsal horn, and also subsequent transmission to the cortex [44]. This mechanism is commonly used in physical medicine treatments, in addition to habituation to a stimulus, and may constitute the basis for forming the hypothesis adopted by the authors of the study reported here about the validity of increasing the strength of the rESWT impact in subsequent treatments.

## 5. Limitations of the Study

Our study has some limitations that may have influenced the obtained results and their interpretation. Patients were included in the study on the basis of a physical examination and various types of imaging-based tests. However, there were no data that would indicate that the subjects had increased vascularization, as indicated by power Doppler ultrasound both before and after the treatment. Apart from refraining from training for the duration of treatment, the authors did not ask the study subjects for more, nor did they monitor the impact of other factors, such as distance covered by walking or time spent standing during the day, over the course of treatment. For ethical reasons, the study did not include a control group of patients not undergoing therapy or staying on sham therapy (to assess the impact of the placebo effect), because all people included in the study experienced pain. The limited sample size entails that the results should not be generalized. Our preliminary results encourage further investigation concerning the impact of changes in shock-wave parameters during treatment depending on the intensity of the pain experienced by the patients.

## 6. Conclusions

The proposed method of treatment, with a gradual increase in the dosage of rESWT, shows greater effectiveness in improving the biomechanical parameters of plantar fascia in a long-term assessment compared to the treatments performed with the application of constant values for the treatment parameters without causing side effects. The proposed method was also more effective for the reduction of the pain experienced.

In our opinion, the presented method of treatment can be used in clinical practice as a safe and effective method of treating patients with plantar fasciitis. Our results are encouraging. The dose escalation in a treatment cycle is worth considering. To prove that this method of treatment is more effective, a randomized controlled trial should be carried out on a representative sample.

## Figures and Tables

**Table 1:** General characteristics of the study participants.

	Group 1 (*n* = 30)	Group 2 (*n* = 32)	*p*-Value
	mean ± SD	mean ± SD	
**Age (years)**	49.86 ± 8.22	52.37 ± 9.81	0.184
**Height (cm)**	170.08 ± 6.92	172.31 ± 7.17	0.544
**Weight (kg)**	70.02 ± 5.48	72.39 ± 6.38	0.462
**BMI (kg/m^2^)**	24.02 ± 2.04	24.45 ± 1.49	0.783
**Length of training history (years)**	9.83 ± 3.61	8.51 ± 3.19	0.475
**Duration of symptoms (month)**	5.04 ± 3.10	4.77 ± 3.92	0.362

**Table 2:** Pain-intensity scores in visual analog scale, VAS, (mean value and standard error of the mean) in both groups, at particular time intervals.

	Before Treatment	After Treatment	1 Month after Treatment	*p*-Value	Effect Size (η2p)
**Group 1**	63.80 ± 15	45.20 ± 16.80	31.40 ± 22.80	<0.001 * 0.003 ** <0.001 ***	0.44
**Group 2**	60.30 ± 18	49.70 ± 15.71	51.40 ± 19.22	<0.001 * 0.587 ** 0.035 ***	0.23
***p*-value**	0.405 ****	0.271 ****	<0.001 ****		
**effect size (Cohen’s d)**	0.21	0.27	0.94		

p*—before treatment vs. right after treatment. p**—after treatment vs. 1 month after treatment. p***—before treatment vs. 1 month after treatment. p****—group 1 vs. group 2.

## Data Availability

The datasets used and/or analyzed during the current study are available from the corresponding author upon reasonable request.
